# Exome analysis of HIV patients submitted to dendritic cells therapeutic vaccine reveals an association of *CNOT1* gene with response to the treatment

**DOI:** 10.7448/IAS.17.1.18938

**Published:** 2014-01-10

**Authors:** Ronald Moura, Alessandra Pontillo, Pio D'Adamo, Nicola Pirastu, Antonio Campos Coelho, Sergio Crovella

**Affiliations:** 1 Department of Genetics, Federal University of Pernambuco, Recife, Brazil;; 2 Department of Immunology, Institute of Biomedical Sciences, University of San Paolo, Brazil; 3 Department of Reproductive Sciences and Development, IRCCS-Burlo Garofolo, University of Trieste, Trieste, Italy

**Keywords:** DC immunotherapy, HIV, exome analysis, *CNOT1*, TTP

## Abstract

**Introduction:**

With the aim of searching genetic factors associated with the response to an immune treatment based on autologous monocyte-derived dendritic cells pulsed with autologous inactivated HIV, we performed exome analysis by screening more than 240,000 putative functional exonic variants in 18 HIV-positive Brazilian patients that underwent the immune treatment.

**Methods:**

Exome analysis has been performed using the ILLUMINA Infinium HumanExome BeadChip. zCall algorithm allowed us to recall rare variants. Quality control and SNP-centred analysis were done with GenABEL R package. An in-house implementation of the Wang method permitted gene-centred analysis.

**Results:**

CCR4-NOT transcription complex, subunit 1 (*CNOT1*) gene (16q21), showed the strongest association with the modification of the response to the therapeutic vaccine (*p=*0.00075). *CNOT1* SNP rs7188697 A/G was significantly associated with DC treatment response. The presence of a G allele indicated poor response to the therapeutic vaccine (*p=*0.0031; OR=33.00; CI=1.74–624.66), and the SNP behaved in a dominant model (A/A vs. A/G+G/G *p*=0.0009; OR=107.66; 95% CI=3.85–3013.31), being the A/G genotype present only in weak/transient responders, conferring susceptibility to poor response to the immune treatment.

**Discussion:**

*CNOT1* is known to be involved in the control of mRNA deadenylation and mRNA decay. Moreover, *CNOT1* has been recently described as being involved in the regulation of inflammatory processes mediated by tristetraprolin (TTP). The TTP-CCR4-NOT complex (CNOT1 in the CCR4-NOT complex is the binding site for TTP) has been reported as interfering with HIV replication, through post-transcriptional control. Therefore, we can hypothesize that genetic variation occurring in the *CNOT1* gene could impair the TTP-CCR4-NOT complex, thus interfering with HIV replication and/or host immune response.

**Conclusions:**

Being aware that our findings are exclusive to the 18 patients studied with a need for replication, and that the genetic variant of *CNOT1* gene, localized at intron 3, has no known functional effect, we propose a novel potential candidate *locus* for the modulation of the response to the immune treatment, and open a discussion on the necessity to consider the host genome as another potential variant to be evaluated when designing an immune therapy study.

## Introduction

Dendritic cell (DC)-based immunotherapy appeared to be an interesting strategy to fight HIV-1 infection and has been proposed firstly as an alternative treatment for HIV-positive (HIV+) patients, then as a co-adjuvant treatment for combined antiretroviral therapy (cART). Indeed, it represents a great chance to investigate the host immune response against the virus. At this time, 11 clinical trials (http://clinicaltrials.gov/) had been concluded and seven are still ongoing [1, 2]. The design of these clinical trials, in terms of antigens used to pulse DC (inactivated whole virus, viral peptides, mRNA, recombinant virus vectors, etc.), DC preparation and dosage, varies between different research groups [1–3]. All DC treatments resulted in being safe and well-tolerated. However, only a few trials showed some results in terms of viral replication control. In particular, the trials conducted by Lu et al. [3] and Garcia et al. [2], implying a similar approach, even if the first enrolled patients without cART treatment while the second considered HIV patients who stopped their cART treatment, appeared to be able to partially control plasma viral load (PVL) for up to 12 or six months, respectively. These findings are the most promising at the moment, but it is interesting to note that only a fraction of patients were able to positively respond to the DC-based treatment.

In the review of Garcia et al. [1], all variables concerning the distinct steps of the immune therapy preparation, clinical trial design and immunological monitoring of treated patients have been deeply considered, proving interesting findings useful for unification criteria of future therapeutic vaccine studies. However, within all the variables considered, the host genome and its influence on the response to therapeutic vaccines have never been taken into account, even if it is well known to affect both HIV-1 replication and AIDS progression [4].

Our research group in 2010 published an article reporting the analysis of innate immunity genome (149 genes and 768 SNPs screened) in the 18 HIV+ patients from the Lu et al. clinical trial [3]. Two SNPs, in *MBL2* and *NOS1* genes, have been associated with better and worse responses to the immune therapy, respectively [5]. In our retrospective analysis, although limited to the innate immunity genome, we evidenced the importance of the host genome as a variable to be considered in the design of a therapeutic vaccine study. Now we are reporting our findings related to the analysis of more than 240,000 exonic variants on the same 18 HIV+ patients, in search for other genetic factors potentially associated with the response to an immune therapy based on autologous monocyte-derived DCs pulsed with autologous inactivated HIV.

## Methods

### Patient's classification

The 18 HIV+ patients who underwent the DC-based treatment were classified as Good Responders (GR) and Weak or Transient Responders (WTR). GR were those who had 90% of PVL reduction after the treatment, as reported in the original study of Lu et al. [3].

### Exome analysis

Genomic DNAs of 18 HIV+ patients from the study of Lu et al. [3] were already available and their quality/quantity was checked at our laboratory. Exome analysis has been performed using the ILLUMINA Infinium Human Exome Bead Chip following manufacturer's protocols. The exonic content consists of more than 240,000 markers with minimum allele frequency>0.05 (mostly cSNPs or few intronic variants known to have been previously associated with clinical phenotypes), representing diverse populations including European, African, Chinese, and Hispanic individuals.

### Data quality control and statistical analysis

Since ILLUMINA exome array has many rare variants, the GenCall (the default ILLUMINA algorithm for genotype calling) could exclude some of them. For this reason, we used the zCall algorithm [6] specifically tailored for rare variants.

The quality control (QC) for the recalling process was performed through the GenABEL [7] package, with a call-rate cut-off set at 99% per SNP call-rate per individual, along with a 95% threshold for identity by state (IBS) and a cut-off of 0.2 for the false discovery rate applied to Hardy–Weinberg equilibrium (HWE) tests. After the QC procedures, 43,636 SNPs (relating to 7,572 genes) passed in all criteria and no individuals were excluded due to low call-rates or any other parameter.

We performed both SNP-centred (also through the GenABEL package) and gene-centred analyses with genomic correction [8]. For gene-based analysis, we used an in-house implementation of the method proposed by Wang & Abbott 2008 [9]. Given the small number of samples in the dataset, *p* values were obtained empirically through a permutation process.

## Results and discussion

The SNP-based approach did not reveal any significant association between the exonic markers and HIV immunotreatment response. Being aware of the limited power of the experiment to detect one single variant association, we performed a gene-based analysis that considered all variants linked as one single piece of information, thus increasing the power of the test. An excerpt of 10 results of gene-centred analysis is reported in Table 1 (the complete results of both SNP and gene-centred tests are available at http://goo.gl/Vb8suZ link).

**Table 1:** Results from gene-centred analysis. The genes are listed by the related *p* value just for illustrative purposes

Gene symbol	Full name	Chr	*p*
*CNOT1*	CCR4-NOT transcription complex, subunit 1	16	0.000026
*CNTN4*	Contactin 4	3	0.000110
*N4BP2L2*	NEDD4 binding protein 2-like 2	13	0.000350
*MANEA*	Mannosidase, endo-alpha	6	0.000410
*OR4E2*	Olfactory receptor, family 4, subfamily E, member 2	14	0.000550
*NHSL1*	NHS-like 1	6	0.000580
*MTA1*	Metastasis associated 1	14	0.001000
*FAIM*	Fas apoptotic inhibitory molecule	3	0.001100
*FGFR2*	Fibroblast growth factor receptor 2	10	0.001420
*ZNF292*	Zinc finger protein 292	6	0.001650

Gene-centred analysis showed that *CNTO1* was the most significantly associated with the response to the immune vaccine. After genetic evaluation we performed a Panther http://www.pantherdb.org/) analysis on the top 10 genes shown in Table 1 in order to investigate if their biological functions were possibly involved in the control of HIV-1 replication.

Within the 10 genes, we selected *CNOT1* because the Panther-based research evidenced a possible involvement in the control of viral replication. *CNOT1* codifies for the core subunit of the CCR4–NOT deanylase complex, which is involved in the control of gene expression through transcriptional regulation and mRNA decay [10, 11]. In our opinion, an altered post-transcriptional process may affect both virion production and host-cell response, as discussed further on.

**Figure 1:**
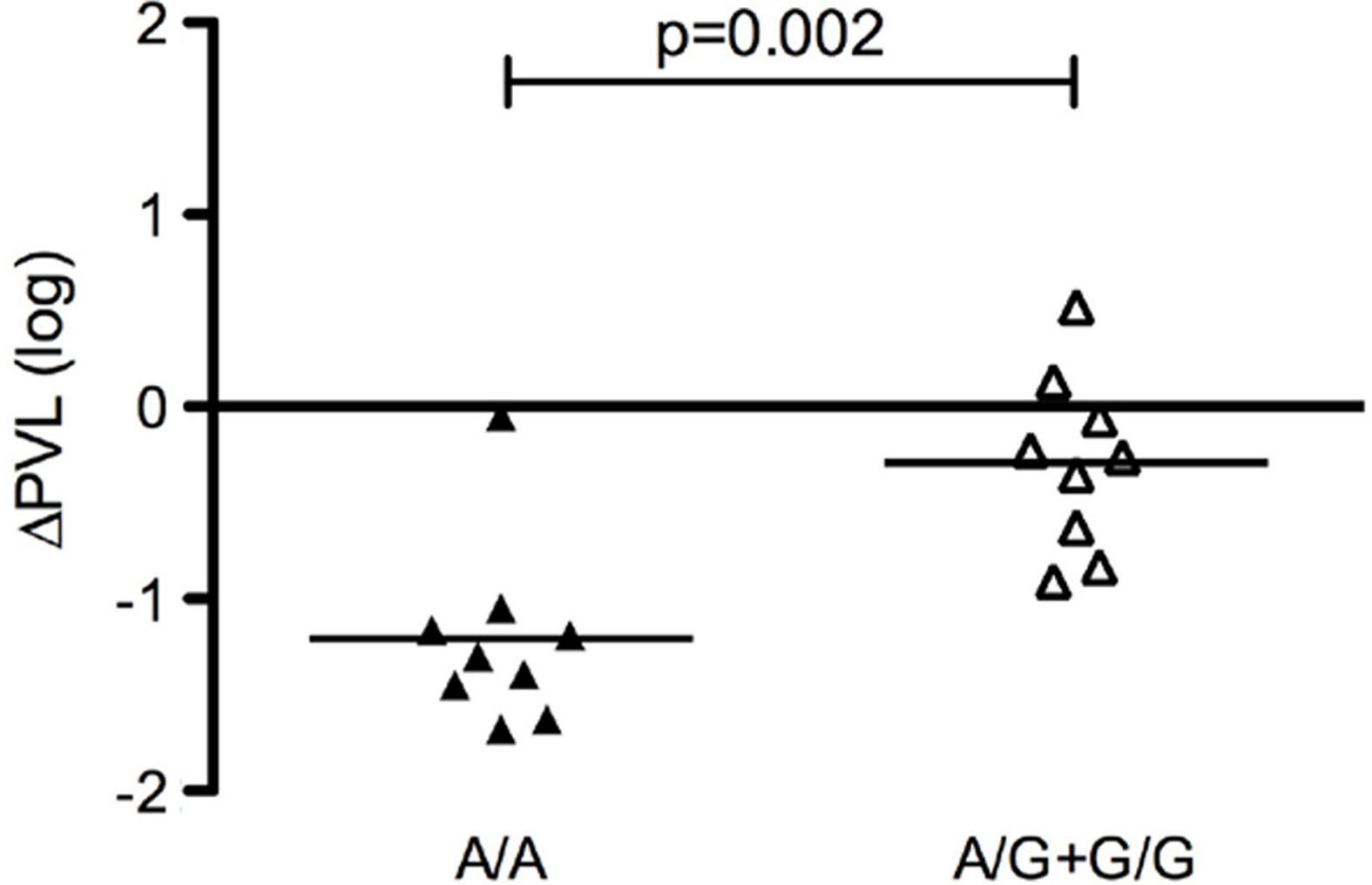
Correlation between *CNOT1* rs7188697 genotypes with PVL changes in the 18 HIV+ patients who have undergone the immune treatment.

Within the 19 SNPs distributed in *CNOT1* gene represented in the ILLUMINA Exome Chip, 15 had fixed monomorphic alleles in our sample while four were polymorphic. Only one SNP, namely the rs7188697 (A>G transition) out of four, showed significant association with DC treatment response. Table 2 reports the different distribution of allele and genotype frequencies of rs7188697 *CNOT1* SNP in GR and WTR patients. The presence of the G allele was strongly associated with poor response to the immune treatment (*p*=0.0031; OR=33.00; 95% CI=1.74–624.66). This was consistent with a dominant model, i.e. A/A vs. A/G+GG (OR=107.66; 95% CI=3.85–3013.31; *p*=0.0009), being the A/G or G/G genotypes present only in WTR patients, conferring high susceptibility to a poor response to the treatment. On the other hand, the A/A genotype, present only in GR subjects, was associated with good response to the immune treatment, as visible in Figure 1.

**Table 2:** Alleles and genotypes counts and frequencies of rs7188697 polymorphism of *CNOT1* gene were reported for the 18 HIV+ patients of the DC-based immune treatment carried out by Lu et al. [3], classified as Weak or Transient Responders (WTR) and Good Responders (GR). Odds ratio (OR), 95% confidence intervals (95% CI) and *p* values from the Chi-squared test for the association between the rs7188697 SNP and the response to the immune treatment. Results for different genetic models are shown

*CNOT1* SNP (rs7188697)	Weak or Transient Responders (WTR) (*n*=10)	Good Responders (GR) (*n*=8)	OR (95% CI)	*p*
**Alleles**				
A	10 (0.5)	16 (1)	Reference	
G	10 (0.5)	0 (0)	33.00 (1.74–624.66)	0.0031
**Genotypes and models**				
Codominant				
A/A	1 (0.1)	8 (1)	Reference	0.0007
A/G	8 (0.8)	0 (0)	96.33 (3.42–2715.42)	0.0015
G/G	1 (0.1)	0 (0)	17.00 (0.46–648.24)	0.4292
Dominant				
A/A	**1 (0.1)**	**8 (1)**	**Reference**	
A/G + G/G	**9 (0.9)**	**0 (0)**	**107.66 (3.85–3013.31)**	**0.0009**
Recessive				
A/A + A/G	9 (0.9)	8 (1)	Reference	
G/G	1 (0.1)	0 (0)	2.68 (0.09–75.12)	0.9084
Overdominant				
A/A + G/G	2 (0.2)	8 (1)	Reference	
A/G	8 (0.8)	0 (0)	57.80 (2.39–1392.38)	0.0035

The bold values indicate the genetic model to be considered.

Furthermore, we evaluated if there was any influence of *CNOT1* genotypes on PVL reported in the work of Lu et al. [3], using the Mann-Whitney test with GraphPad Prism software. For this test, a *p*<0.05 was considered statistically significant. The *CNOT1* rs7188697 genotypes also correlated with PVL changes in the 18 HIV+ patients, since only individuals with A/A genotype showed significant reduction of PVL.

After genetic analysis, we also performed an *in silico* evaluation of the possible interactions between the proteins encoded by the 10 selected genes (see Table 1) using the GeneMania Software (http://www.genemania.org/). We searched for possible boundaries prediction between CNOT1 and the proteins encoded by the other nine genes, without finding any common pathway or predicted interaction between them.

When considering the role of CNOT1 in the process of HIV-1 replication, this protein appeared to be important both in immune response as well as in HIV-1 replication. Paul et al. [12] suggested that CNOT1 is involved in the transcriptional regulation of the major histocompatibility complex (MHC) class II transactivator (CIITA) and MHC-II expression linking CNOT1 with antigen presentation. Moreover, CNOT1 has been recently described to interact with tristetraprolin (TTP), an RNA-binding protein able to regulate and control the inflammatory response with a mechanism involving the reduction of the expression profile of several inflammatory cytokines [13].

Conversely, TTP was reported to be able to inhibit HIV-1 replication by directly binding viral genome interfering with RNA post-transcriptional mechanisms [14].

With all of this considered, we might hypothesize that CNOT1 could affect immune response against HIV-1 indirectly through the transcriptional control of MHC-II and inflammatory genes, and possibly directly interact with TTP in the inhibition of virus replication. In this way, genetic variation occurring in the *CNOT1* gene, identified as a potential candidate for poor response to the therapeutic vaccine, could impair the TTP-CCR4-NOT complex, thus interfering with mRNA stability of HIV-1 RNA and inflammatory transcripts, promoting an increased production of viral particles and inflammatory status, both important factors of poor prognosis of HIV-1 infection. Moreover, high HIV-1 replication rates and chronic inflammation have been described as potentially affecting HIV+ DCs manipulation and anti-HIV therapeutic vaccine response [15–18].

**Figure 2:**
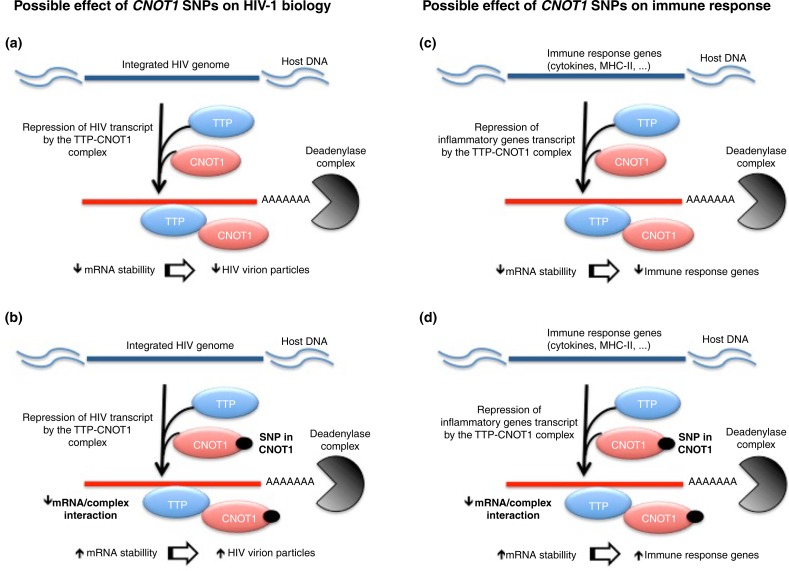
Hypothesis for *CNOT1* involvement in anti-HIV DC-based immune treatment response. (a) CNOT1 could interact with TTP leading to the repression of HIV transcripts. (b) SNPs in CNOT1 could affect TTP and/or mRNA binding augmenting HIV RNA stability and the consequent production of HIV virions. (c) CNOT1 could, directly or interacting with TTP, inhibit the expression of immune genes (such as cytokines and MHC-II) and consequently the immune response. (d) SNPs in *CNOT1* could affect mRNA binding augmenting mRNA stability and the consequent expression of immune genes, leading to an increased immune response. CNOT1: CCR4-NOT complex; TTP: tristetraprolin; SNPs: single nucleotide polymorphisms. ↑=increase; ↓=decrease.

Figure 2 establishes a mechanism on how CNOT1 would be involved in the control of viral replication and immune response.

The rs7188697 *CNOT1* SNP, associated with WTR, is localized at intron 3 of the gene and, to our knowledge, has no functional implication in the regulation of *CNOT1* expression. For this reason, we are cautious in the interpretation of our findings and aware that no study until now has aimed at disclosing a possible association of *CNOT1* genetic variations and susceptibility to HIV infection or virus replication.

With the aim of disclosing if rs7188697 *CNOT1* SNP could be a potential genetic marker, we investigated the distribution of its allele frequencies in the ethnic groups described in NCBI SNPs database (http://www.ncbi.nlm.nih.gov/projects/SNP/snp_ref.cgi?rs=7188697): the G allele, strongly associated with poor response to the immune treatment, is well represented and distributed in the HapMap-CEU (European, A=0.691; G=0.309), HapMap-HCB (Asian Chinese, A=0.464; G=0.536), HapMap-JPT (Asian-Japanese A=0.663; G=0.338) and HapMap-YRI (Sub-Saharan African A=0.840; G=0.160) populations. Being quite frequent with a minimum allele frequency (MAF) >16% (YRI) in all of the HapMap populations screened, we can consider rs7188697 SNP as a well-represented and potential genetic marker.

Since our study is retrospective and only genomic DNA from the 18 HIV+ patients of Lu et al. [3] was available, we were not able to perform any functional study (i.e. evaluation of *CNOT1* gene expression) to confirm our hypothesis. Moreover, our findings have been obtained just on patients from a single phase I trial such as the one of Lu et al. [3], so they should be considered as “exclusive to the study” and strongly need to be replicated in other DC vaccine patients from other clinical trials.

## Conclusions

Our findings propose a novel, although exclusive to the phase I study of Lu et al. [3], potential candidate, *CNOT1*, for the modulation of the response to the therapeutic vaccine and do open a discussion on the need to consider the host, i.e. a genome, as another variant likely to be evaluated when designing an immune therapy study, or to be retrospectively analysed once the study is completed. With this paper, being aware of the limitations of our findings obtained on a low number of patients and missing functional validation of the genetic association because of lack of biological material, we would like to recall the attention of other research teams joining the workshop on DC-based vaccine clinical trials group [1]. We would encourage them to create a genetic network aimed at analysing the genomes of HIV patients treated with DC-based immune therapy in order to provide new insights on the role of the host genome in the response to immune treatments.
